# The multiple roles of GH in neural ageing and injury

**DOI:** 10.3389/fnins.2023.1082449

**Published:** 2023-03-07

**Authors:** Daniel G. Blackmore, Michael J. Waters

**Affiliations:** ^1^Queensland Brain Institute, The University of Queensland, Brisbane, QLD, Australia; ^2^Clem Jones Centre for Ageing Dementia Research, Queensland Brain Institute, The University of Queensland, Brisbane, QLD, Australia; ^3^Institute for Molecular Bioscience, The University of Queensland, Brisbane, QLD, Australia

**Keywords:** growth hormone, ageing, cognition, neural function, neural stem cells, exercise

## Abstract

Advanced age is typically associated with a decrease in cognitive function including impairment in the formation and retention of new memories. The hippocampus is critical for learning and memory, especially spatial learning, and is particularly affected by ageing. With advanced age, multiple neural components can be detrimentally affected including a reduction in the number of neural stem and precursor cells, a decrease in the formation of adult born neurons (neurogenesis), and deficits in neural circuitry, all of which ultimately contribute to impaired cognitive function. Importantly, physical exercise has been shown to ameliorate many of these impairments and is able to improve learning and memory. Relevantly, growth hormone (GH) is an important protein hormone that decreases with ageing and increases following physical exercise. Originally described due to its role in longitudinal growth, GH has now been identified to play several additional key roles, especially in relation to the brain. Indeed, the regular decrease in GH levels following puberty is one of the most well documented components of neuroendocrine ageing. Growth hormone deficiency (GHD) has been described to have adverse effects on brain function, which can be ameliorated *via* GH replacement therapy. Physical exercise has been shown to increase circulating GH levels. Furthermore, we recently demonstrated the increase in exercise-mediated GH is critical for improved cognitive function in the aged mouse. Here we examine the multiple roles that GH plays, particularly in the aged brain and following trauma, irradiation and stroke, and how increasing GH levels can ameliorate deficits in cognition.

## Introduction

It has long been known that the pituitary gland is critical for growth. In 1912, Cushing postulated the existence of a “hormone of growth” within the pituitary gland. Experiments in the 1920s, in which intraperitoneal injections of bovine pituitary gland extracts increased the growth of rats (Evans and Long, 1922; Johnson and Everett, 1929), confirmed this hypothesis. The discovery and successful purification of growth hormone (GH), however, took several subsequent decades starting with the isolation of oxen GH in 1944 (Li and Evans, 1944) and culminating with the purification of human GH (hGH) in 1956 (Li and Papkoff, 1956). Within 2 years of this discovery, the positive effect of GH treatment on human growth was reported (Raben, 1958). Treatment of patients with purified hGH required obtaining human pituitary glands and therefore GH therapy was reserved for the most severe cases of GH deficiency (GHD) (Ayyar, 2011). The identification of the hGH sequence in the 1960s (Li et al., 1966, 1969) and the sequence of bovine GH soon after (Graf and Li, 1974), allowed for the successful cloning and expression of hGH, thereby allowing a wider range of individuals to be treated with GH. This was followed by the cloning of the receptor itself and the elucidation of its signalling processes (reviewed in Waters, 2016).

The availability of pure hGH made it possible to measure circulating hGH levels by immunoassay, beginning in the 1960s. The pulsatile release of GH was then documented by Tannenbaum and Martin (1976). It was discovered that both males and females had multiple GH peaks throughout the day and night with young females possessing the highest peaks (Ho et al., 1987). Soon after, it became apparent that circulating levels of GH changed throughout a person’s lifetime with peak GH levels reached at puberty for both genders. In both genders, this is followed by an age-dependent decrease in both general baseline levels and pulsatile peak heights (Ho et al., 1987). It is now accepted that peak levels of GH decrease approximately 10–15% every consecutive decade beyond the age of 30 (Garcia et al., 2019). This decrease, coupled with a general decrease in metabolism and body composition, is collectively referred to as Somatopause (Lanfranco et al., 2003; Roelfsema and Veldhuis, 2016). Individuals over 60 have been routinely diagnosed as being GHD. Those suffering from adult onset GHD typically have deficits in body composition, cognitive processing and general wellbeing (Sattler, 2013) compared to age-matched controls.

Concurrent with the age-dependent decrease in GH levels is an age-associated decrease in certain domains of cognitive ability. The formation and retention of new memories, especially those related to spatial learning, appear to be especially sensitive to ageing (Gallagher and Pelleymounter, 1988). The hippocampus is a region of the brain critical for a range of cognitive processes including learning and successful retention of memories (Lisman et al., 2017). It is also one of two main regions in the mammalian brain that continue to generate new neurons during adulthood in a multi-step process referred to as neurogenesis (Eriksson et al., 1998). Neurons generated in the dentate gyrus of the hippocampus are critical for spatial learning (Vukovic et al., 2013). The second region that continues neurogenesis into adulthood is the subventricular zone (SVZ) of the lateral ventricle. Immature neurons generated in the SVZ migrate to the olfactory bulb where they integrate and contribute to olfaction. Both brain regions are detrimentally affected with advanced age, resulting in deficits at each stage of neurogenesis including; a reduction in the number of activated neural stem cells (NSCs) (Walker et al., 2008; Blackmore et al., 2009), a decrease in adult born neurons (Blackmore et al., 2021), deficits in cognitive function including spatial learning (van Praag et al., 2005) and reduced fine olfactory discrimination (Enwere et al., 2004).

Physical exercise is one of the strongest positive stimulators of both cognitive function and circulating GH. The beneficial effects of physical exercise on the brain, especially in relation to hippocampal neurogenesis and spatial learning, was originally discovered in adult mice by the seminal work of van Praag et al. (1999a,b). This was then extended to aged mice some years later (van Praag et al., 2005). Similarly, physical exercise has been described to be beneficial to human cognition in both young (Winter et al., 2007) and aged individuals (Cotman and Berchtold, 2002). Importantly, physical exercise has also been clearly demonstrated to increase circulating GH levels. Studies in humans reported that exercise-mediated increases in GH levels (Sutton and Lazarus, 1976) are exercise intensity-dependent (Felsing et al., 1992; Pritzlaff et al., 1999). Importantly, exercise, even in individuals of advanced age, continues to mediate increases in GH levels of both males and females (Giannoulis et al., 2005; Weltman et al., 2006). Similarly, in rodents, it was found that GH levels increased in the rat following treadmill exercise (Bigbee et al., 2000). We recently confirmed comparable results in adult and aged mice (Blackmore et al., 2021).

Even though there are coincident decreases in GH levels and cognitive function during ageing, and both respond positively to exercise, it was originally believed that GH affected peripheral tissues exclusively and not the brain. Therefore, any effect of GH on the brain was assumed to be indirect. Work in the late 1970s and early 1980s began to alter this view with the finding that intravenous injection of rGH in hypophysectomised rat resulted in decreased catecholamine secretion from the hypothalamus (Andersson et al., 1977, 1983). The finding that the GH receptor (GHR) is expressed in the rodent (Lobie et al., 1993) and human (Castro et al., 2000) brain and the demonstration that GH is transported into the brain (Pan et al., 2005), further established the potential for a direct role of GH mediating effects within the brain. It is now well established that GH directly affects several components within the brain, including stem cell activation and neural plasticity. This manifests as altered brain structure in children with isolated GHD, notably in reduced hippocampus, thalamus, and globus pallidum volumes as assessed by MRI (Webb et al., 2012). Ultimately these GH-mediated changes culminate in improvement of some components of cognitive ability in GH replacement models of GHD and clinical GHD, particularly those related to fluid memory to which spatial learning belongs. There is also an improvement in processing speed and movement-associated battery scores in isolated GHD children receiving GH replacement (Webb et al., 2012). It should be noted that adult-onset GHD is not limited to disorders of the pituitary or physiological age but can also be induced through injury including stroke or following trauma including whole brain irradiation. Here we review the effects of manipulating GH, either naturally or pharmacologically, within the brain with particular reference to age and injury. It should be noted that the effect of altering GH levels is often described interchangeably with changes in the GH/IGF-1 axis. This is because IGF-1 was originally believed to be responsible for mediating all of GH actions, a view that has since altered to include direct effects of GH (Sonntag et al., 2005). This original assumption was likely because IGF-1 can pass through the blood–brain barrier (BBB) and has also been shown to enhance NSC proliferation, neurogenesis (Aberg, 2010), and improve cognitive function (Aleman et al., 1999). For the purposes of this review, the focus will be on the direct effect of GH within the brain.

## GHR^+ve^ cells are present within the central nervous system and include neural stem cells

The first identification and mapping of murine GHRs within the brain was published in 1993 (Lobie et al., 1993). This finding was surprising as prior to this, the role of GH was considered to involve only peripheral tissues. Notably, this study found that GHRs were strongly expressed during neurogenesis and decreased subsequently, intimating at a potential role during brain development. Consequent studies also identified the presence of GHR within embryonic rat cerebral cortical cells (Ajo et al., 2003) and the dentate gyrus of the adult rat hippocampus (Fujikawa et al., 2000). GHR was identified within neural tissue of other species including chicken (Harvey et al., 2002) and humans (Castro et al., 2000). The first indication that GH is capable of crossing the BBB came when adult-onset GHD patients treated with recombinant human GH (rhGH) demonstrated a dose-dependent increase of GH within the CSF (Johansson et al., 1995; Burman et al., 1996). Confirmation of GH influx across the BBB of rodents was then achieved by measuring ^125^I-labelled rat GH in brain homogenates of both mice and rats (Pan et al., 2005). This finding further solidified the potential for a direct role of GH within the central nervous system (CNS). Of note, Pan et al. (2005) ruled out a specific active transport system for GH as they were unable to identify a saturable active transport system. Interestingly, whilst the majority of GH is synthesised within the anterior pituitary gland, it is now established that GH is also synthesised within non-pituitary regions of the brain of birds (Ramesh et al., 2000) and rodents (Donahue et al., 2006), including the Ames dwarf mouse that is deficient in pituitary-derived GH (Sun et al., 2005). The finding of extrapituitary GH in the brain supports a role for a GH brain axis separate from the periphery but does not exclude input from circulating pituitary GH through its uptake *via* the BBB.

In order to determine the identity of neural GHR^+ve^ cells, we subsequently used FACS to isolate these cells from the SVZ of the lateral ventricles of adult mice (Blackmore et al., 2012a). This region of the lateral ventricles has been demonstrated to be the primary location of SVZ neural stem and precursor cells (Lois and Alvarez-Buylla, 1994). *In vitro* culture of these cells confirmed the ability of GHR^+ve^ cells to display the main characteristics attributed to stem cells (Gage, 2000): form neurospheres, undergo long-term proliferation and become multipotent upon differentiation (Blackmore et al., 2012a). More recently we showed that the GHR transcript was enriched in Nestin-GFP^+ve^/EGFR^+ve^ precursor cells purified from the adult murine hippocampus (Blackmore et al., 2021). Together, these results demonstrate that GHR is expressed within the adult brain, including in neural precursor cell populations from the two main neurogenic regions of the brain. It is interesting to note that, as with the decrease in GH with advancing age, reports demonstrate age-dependent decreases in GHR density within the human brain (Lai et al., 1993) and throughout the CNS of rats, including the hippocampus (Lobie et al., 1993; Zhai et al., 1994).

## GH *in vitro* and *in vivo* increases stem and precursor cell number in adult and aged rodents

Given GH’s known role regarding the growth of internal organs and peripheral tissue during development, it is perhaps not surprising that GH has also been demonstrated to be involved in the induction of embryonic NSC proliferation. Initial experiments conducted on rat embryonic cerebral cortical tissue demonstrated that addition of GH increased proliferation of cortical precursors (Ajo et al., 2003). Similarly, *in vitro* GH treatment increased the rate of proliferation in passaged embryonic mouse SVZ neurospheres (Christophidis et al., 2009). The ability of GH to increase the rate of proliferation in NSCs was confirmed for humans using human embryonic-derived NSCs (Pathipati et al., 2011). Addition of GH to adult, wild-type (WT) SVZ primary cultures both increased neurosphere number and proliferation rate (McLenachan et al., 2009), demonstrating that GH continues to activate NSCs in adult neural tissue. In these studies, however, unsorted primary neural tissue was used to examine the effect of GH on stem cell proliferation. It was therefore important to determine if GH directly activated NSC populations by examining the effect of GH on purified subpopulations of NSCs.

We therefore took advantage of our sub-populations of neural precursor and stem cells that expressed GHR^+ve^ purified from both the SVZ and hippocampal regions of adult WT mice (Blackmore et al., 2012a,^2021^) to examine this. *In vitro* addition of GH to purified adult SVZ GHR^+ve^ cells increased neurosphere number and expansion rate during long-term serial passaging, indicating that GH directly activated NSCs (Blackmore et al., 2012a). Direct activation by GH was confirmed when primary SVZ tissue was cultured *in vitro* and the N-CFC assay was used to identify stem cell-derived colonies based on size (Reynolds and Rietze, 2005). A higher number of large, stem cell-derived colonies followed the addition of exogenous GH (Blackmore et al., 2012a). Purified, clonal adult hippocampal Nestin-GFP^+ve^/EGFR^+ve^ precursor cells were also found to be responsive to *in vitro* GH treatment (Blackmore et al., 2021). Taken together, these results indicate that GH can act directly on NSCs in adult brain samples.

Unfortunately, during ageing, there is a dramatic decrease in NSC number within both the hippocampus (Walker et al., 2008) and SVZ (Blackmore et al., 2009), making it technically impossible to obtain purified stem cell populations from aged brains on which to test directly the effect of GH *in vitro*. However, unsorted neurosphere cultures from aged tissue confirmed that ageing NSCs retain their ability to be activated with GH treatment in both the SVZ and hippocampus (Blackmore et al., 2012b,^2021^). Interestingly, similar to the ageing brain, there is also a marked decrease in stem cell number in GHR knockout (GHRKO) mice (McLenachan et al., 2009; Deleyrolle et al., 2011; Blackmore et al., 2012a). This decreased number of stem cells may contribute to the decreased size of brains from GHRKO animals compared to WT littermates. SVZ precursor cells from GHRKO animals possess a reduced neurosphere size (McLenachan et al., 2009), a decrease in passaged proliferation rate (Blackmore et al., 2012a), and as one would expect, do not respond to exogenous GH in *in vitro* culture (Blackmore et al., 2012b).

As described above, whilst adult GHRKO animals show a decreased proportion of NSCs, they do retain a population of stem cells. Similarly, inhibition of GH action *in vitro via* addition of a GH antagonist to adult (Blackmore et al., 2012b; Devesa et al., 2014) and aged brains (Blackmore et al., 2021) reduces precursor cell number to non-activated, control levels. This suggests that GH activates a subpopulation of endogenous neural precursor cells. It appears that at least two distinct sub-populations of precursor cells reside within the adult brain that can be activated by alternative stimuli, a finding that was confirmed within the hippocampus of adult mice (Jhaveri et al., 2015).

Having demonstrated that GH activates NSC proliferation in embryonic, adult and aged primary tissue using *in vitro* tissue culture techniques, attention moved to the effect of exogenous GH treatment on proliferation *in vivo*. One of the first studies to address this demonstrated that a 10-week, twice daily subcutaneous (s.c.) injection of rhGH significantly increased the number of hippocampal neurons in aged Wistar rats (Azcoitia et al., 2005). However, in this study, it was not possible to determine if GH treatment altered proliferation rates or increased neuronal survival. This was subsequently addressed when adult hypophysectomised rats were injected s.c. daily with bovine GH (bGH) for 6 or 28 days in combination with intraperitoneal BrdU injections (Aberg et al., 2009). This approach was undertaken to remove the confound of pituitary derived GH and to induce GHD in the rat. Interestingly, both injection paradigms successfully increased the number of BrdU^+ve^ cells in the hippocampus, however, only the longer, 28-day bGH treatment increased proliferation within the SVZ. This suggests differences in precursor cell activation timing between these two neurogenic regions. Subsequently, Aberg et al. (2010) also examined the effect of peripheral bGH treatment on neural proliferation in the intact adult rat brain. In this instance, a 5-day s.c. injection paradigm induced a doubling in BrdU^+ve^ cells within the dentate gyrus, thus demonstrating that GH supplementation can augment hippocampal cell proliferation above normal levels. To examine the direct effect of GH supplementation, we conducted intercranial ventricular (ICV) infusion of GH into adult (Blackmore et al., 2012a) and aged (Blackmore et al., 2012b) WT mice. In both instances, we observed significant increases in NSC activation as measured by increased neurosphere number. Interestingly, we also noted that an acute, 7-day infusion appeared to increase the number of NSCs for at least 120 days (Blackmore et al., 2012a), suggesting a reprogramming of stem cell activation status. This may occur by altering the methylation or epigenetic status within these cells, as was recently demonstrated in a human study in which a combinational injection paradigm that included GH, DHEA and metformin reversed the epigenetic age of a small cohort of human subjects (Fahy et al., 2019).

## Control of GH release during ageing

Control of GH secretion is complex. It continues to be the focus of extensive research and has been comprehensively addressed in multiple excellent reviews (Muller et al., 1999; Pombo et al., 2001). Contributing to the complexity of GH release are the physiological components of ageing, which continue to be elucidated. The age-related decrease in circulating GH levels is the culmination of several inter-connected cascading events, with the main components including a change in: (1) the relative abundance, sensitivity and responsiveness between the two main positive and negative regulators of GH secretion, GH releasing hormone (GHRH) and somatostatin (SRIF) respectively; (2) an age-dependent change in the principal form of SRIF; and (3) morphological changes to the pituitary gland (Console et al., 1993).

Whilst there is little change in the frequency of GH pulsatile release, the pulse amplitude is dramatically reduced during ageing for both genders (Ho et al., 1987). Induction of a GH pulse requires appropriate timing of upstream regulatory components, which include both the release of GHRH and an inhibition of somatostatin tone (Hindmarsh et al., 1991). Early work demonstrated an age-dependent decrease in the number of GHRH producing neurons and GHRH mRNA levels within the hypothalamus of ageing rats (DeGennaro Colonna et al., 1989). A decrease in GHRH was therefore considered to contribute to the reduced amount of GH being secreted with advanced age.

A change in somatostatin level was also touted as being the predominant mechanism by which GH levels were reduced. Initial experiments showed that the pharmacological induction of GH release was significantly blunted in aged rats (Sonntag et al., 1981). Co-injection of somatostatin antiserum, however, resulted in comparable secreted GH levels between adult and aged male rats, leading the authors to two possible conclusions – the first being that SRIF becomes more abundant during ageing whilst the second was that the pituitary becomes more sensitive to SRIF (Sonntag et al., 1981). Subsequent work by this group appeared to point to the latter as induction of GH release was reduced in aged rats *in vivo*. However, when pituitary gland slices from adult and aged rats were removed and treated *in vitro* with synthetic GH releasing factor, similar levels of secreted GH were obtained from the two preparations following stimulation (Sonntag et al., 1983), a finding that demonstrated an increased influence of hypothalamic control of GH release during ageing. Interestingly, whilst the concentration of basal levels of SRIF remain constant during ageing (Sonntag et al., 1986), the relative concentrations of the two main isoforms, somatostatin-14 and somatostatin-28 (SRIF-14 and -28, respectively), do not. Following the discovery of multiple isoforms of SRIF (Meyers et al., 1980; Patel et al., 1981), Tannenbaum et al. (1982) showed that SRIF-28 is longer lasting on the pituitary. Soon after, Sonntag et al. (1986) demonstrated an age-dependent increase in the prevalence of SRIF-28 in male rats. Taken together, it appears that SRIF, especially the age-associated SRIF-28 isoform, plays a key role in the diminution of GH secretion from the pituitary gland during ageing. This is compounded by significant age-dependent decreases in both the number and volume of individual somatotroph cells within the pituitary gland (Console et al., 1993), a finding we recently confirmed in ageing WT mice (Blackmore et al., 2021).

As can be seen from above, the aetiology of an age-dependent decrease of GH secretion is multifactorial. A decrease in the presence of GHRH-containing neurons in the hypothalamus (DeGennaro Colonna et al., 1989), coupled with an increase in the more potent bioactive, age-associated SRIF-28 somatostatin isoform (Tannenbaum et al., 1982), likely contribute to morphological changes observed in the pituitary gland (Console et al., 1993). This culminates in a significant decrease in GH secretion, which has been demonstrated to have multiple deleterious effects in physiological ageing. Furthermore, as described above, there is also a decrease in the distribution of GHRs within the aged brain (Lobie et al., 1993; Zhai et al., 1994). This constellation of events is collectively referred to as somatopause. Importantly however, even at an advanced age, it is still possible to stimulate GH release naturally with physical exercise being one of the most effective physiological approaches by which to achieve this.

## The exercise-mediated response of GH secretion is altered in ageing

Slowing the age-dependent decrease in GH under physiological conditions has been shown to improve multiple health parameters. These include a change in body composition, improved heart health, metabolism and importantly, altered cognitive health in terms of mood and enhancement of multiple components of learning and memory. One of the most effective ways to increase GH secretion is *via* physical exercise. Some of the earliest work noted that aerobic physical exercise increased GH levels in humans in an intensity-dependent manner (Sutton and Lazarus, 1976). Indeed, as testament to the effectiveness of exercise increasing GH levels, this approach surpassed the pharmacological induction of GH release induced by arginine and L-DOPA (Sutton and Lazarus, 1976). Continued research in this field proposed key components that govern the amount of GH released during and immediately after physical exercise. These include gender (Pritzlaff-Roy et al., 2002), obesity (Kanaley et al., 1999), physical fitness (Holt et al., 2001), exercise intensity (Felsing et al., 1992; Pritzlaff et al., 1999), and age (Hagberg et al., 1988; Craig et al., 1989a,b; Pyka et al., 1992).

To better understand the age-associated diminution of GH responses to exercise, research groups began to interrogate further these potential regulatory components. For example, some have shown that increased adiposity can result in a significant attenuation of exercise-mediated GH release in young adult men (Weltman et al., 2001) and women (Kanaley et al., 1999). However, another group found that under comparable exercise conditions, overweight young male adults retained the ability to increase GH levels (Holt et al., 2001). In contrast, aged overweight males showed a reduced GH response to exercise compared to age-matched, lean individuals. Using multivariate analysis, these authors determined that age, and not adiposity, was the main determining factor in the decreased response. They also determined that during ageing, maintenance of physical fitness as assessed by VO_2_ max, is also critical to the preservation of GH secretion levels (Holt et al., 2001). Interestingly, endurance training, which can improve physical fitness, significantly enhances GH release after 1 year of training in young adults, especially when exercise is conducted above the Lactate threshold (Weltman et al., 1992). This response is blunted relatively early. Middle-aged men following a 4-month progressive endurance training paradigm exhibited a significant age-related decline in exercise-mediated GH secretion levels that was not enhanced following extended training (Zaccaria et al., 1999). Directly comparing gender and ages during a graded exercise testing paradigm similarly revealed an overall decrease in GH response in the aged participants (Weltman et al., 2006). This study also found no gender differences in response to exercise for the aged participants and that only the highest intensities of exercise resulted in statistically significant elevations in GH levels (Weltman et al., 2006). The latter decreased sensitivity to exercise suggests that ageing may increase the degree of inhibition of exercise-mediated GH release.

As discussed earlier, it is known that somatostatin, particularly the age-associated form, SRIF-28 (Sonntag et al., 1986), is a potent inhibitor of GH release from the pituitary. It is upregulated in response to raised GH levels (Giustina and Veldhuis, 1998). Previous studies have also shown that GH release in response to exercise is mediated by lowered somatostatin release (Thompson et al., 1993). To directly examine the influence of SRIF on GH release, Marcell et al. (1999) inhibited SRIF during exercise in adult and aged individuals. They found that administration of pyridostigmine bromide (PYR), an acetylcholine esterase inhibitor that inhibits SRIF secretion (Muller, 1997; Obermayr et al., 2005), enhanced the GH response to exercise in both adult and aged males. Whilst PYR administration in the aged participants significantly increased exercise-mediated GH secretion, it did not restore GH to youthful levels. This finding led the authors to conclude that additional components beyond somatostatin are involved in the age-dependent decrease in GH secretion following exercise.

Of note, studies examining the effect of exercise on GH levels in aged individuals also revealed that the exercise-induced increase in GH seen in aged individuals occurs later than in young adults (Marcell et al., 1999). Similarly, we recently showed that in young mice, GH levels are increased following acute periods of exercise whilst a prolonged period of voluntary wheel running was required to increase GH levels both in the blood and the pituitary gland of 24-month-old mice (Blackmore et al., 2021). This exercise-mediated increase in GH coincided with morphological changes in the pituitary gland, including an increase in both the total number of cells and the density of GH-containing cells in the pituitary of 24-month-old mice. A coincident decrease in SRIF levels was also observed in these animals. We found that pharmacological treatment to reduce somatostatin tone using donepezil, an acetylcholine esterase inhibitor, extended circulating GH levels during prolonged exercise (Blackmore et al., 2021). Finally, as with human studies (Weltman et al., 2006), we noted that a lower exercise intensity, achieved by using low resistance running discs, failed to increase GH levels in aged mice (Blackmore et al., 2021).

The optimal increase in exercise-mediated GH levels in adult and aged mice also coincided with an increase in NSCs. Indeed, we and others have found that optimisation of exercise to increase NSCs appeared to be dependent on age, with younger animals requiring acute exercise (Blackmore et al., 2009; Overall et al., 2013; Leiter et al., 2022) and older animals requiring a prolonged exercise duration (Kohman et al., 2012; Blackmore et al., 2021; Zhou et al., 2021). To determine if the exercise-mediated increase in stem cell number was due to increased GH activation, we directly infused competitive GH antagonists during exercise and found NSC number to remain at sedentary, non-run levels in SVZ and hippocampal neurogenic regions (Blackmore et al., 2012b,^2021^).

It is clear that advanced age results in decreased circulating GH levels, especially GH peaks, and physical exercise retains its ability to increase GH levels in aged humans and animals. Whilst peak GH levels in the aged fail to reach youthful levels, they appear to be sufficient to improve several measures of health and wellbeing, including measures of cognitive function. Animal models have further elucidated the mechanisms involved in these processes and shed light on how manipulation of GH levels contributes to improved learning and memory in those suffering from GHD, during ageing and following traumatic brain injury (TBI), stroke, and whole-brain irradiation therapy.

## GH improves cognitive function in models of GHD, during ageing and following neural injury

As stated above, animal models have been intrinsic to understanding the effect of GH on cognitive function. Induction of GHD, either in spontaneous GHD rats (Li E. et al., 2011) or more typically *via* hypophysectomising animals, robustly induces cognitive deficits in rodents. These deficits, typically expressed as decreased spatial learning ability, can be reversed *via* GH administration (Le Grevès et al., 2006; Ramis et al., 2013). In parallel with animal studies investigating the effect of GHD and GH replacement therapy on cognition, clinical studies also examined this as a potential therapeutic approach. A relationship between cognitive measures and those suffering from GHD in humans was established by the early work of Deijen et al. (1996). They compared differences in psychological complaints between adult males suffering from GHD as a result of either isolated growth hormone deficiency (IGHD) or multiple pituitary hormone deficiency (MPHD). This wide-ranging study identified that MPHD subjects possessed a more severe deficit in GH secretion than IGHD patients. The MPHD subjects also displayed a wider range of psychological issues including decreased vigour and self-esteem whilst also possessing higher anxiety levels. These psychological issues were primarily attributed to deficiencies in hormones, including testosterone and ACTH. Both MPHD and IGHD groups, however, also displayed deficits in specific domains of cognitive function including iconic, short-term and long-term memory (Deijen et al., 1996). The effect of GH supplementation on psychological and cognitive outcomes was assessed in adult male GHD patients subjected to a 2-year GH replacement therapy regimen (Deijen et al., 1998). This study demonstrated both a time-dependent and GH-dose response effect in improving specific components of cognitive function including short-term and long-term memory abilities. Patients receiving a supraphysiological GH dose displayed a more rapid improvement in cognitive function than those receiving lower GH doses. However, both cohorts showed significant improvements after 12 months of GH treatment, improvements that were maintained for the subsequent 12 months (Deijen et al., 1998). Similar results were found by Soares et al. (1999) who showed a 6-month treatment of rhGH improved measures of attention, cognitive efficiency, comprehension and vocabulary in adults suffering from GHD. Importantly, the long-term benefits of GH replacement for GHD patients appear to be stable with a longitudinal study showing prolonged positive outcomes in health parameters and cognitive function (Arwert et al., 2005). Indeed, both short-term and long-term associative tasks remained significantly improved 10 years following initiation of GH replacement. More recently it was shown that an acute, 3-week period of GH treatment of an elderly female suffering from cognitive decline was sufficient to improve learning ability. A PET-SCAN revealed a normalisation of cerebral metabolism within the hippocampus (Devesa et al., 2018).

It should be noted that side effects from GH replacement therapy have been commonly reported and include fluid retention (Lanfranco et al., 2003), peripheral oedema and carpel tunnel syndrome (Hersch and Merriam, 2008). Accordingly, researchers altered their experimental approach to induce a more physiological release of GH *via* GHRH and GHRH-analogue treatments, especially in instances of adult onset GHD or physiological ageing. Initial work in the aged rat demonstrated that GHRH administration is effective at attenuating age-related deficits in spatial memory, especially during the probe portion of Morris water maze (MWM) trials (Thornton et al., 2000). In humans, treatment with GHRH and analogues typically resulted in both a lowered incidence and severity of any reported side effects compared to GH replacement therapy (Merriam et al., 2003). Examining the ability of GHRH treatment to overcome the age-dependent decrease in GH in aged individuals typically revealed improvements in specific domains of cognitive function, especially those related to areas of fluid intelligence such as comprehension and reasoning. For example, a 6-month treatment with GHRH in healthy aged participants resulted in improved cognitive domains that included problem solving and working memory (Vitiello et al., 2006). Importantly, this latter study also demonstrated comparable improvements for both males and females, highlighting the utility of such an approach in aged individuals. The clinical importance of GH levels in physiologically aged individuals is further highlighted by the fact that GH levels induced *via* GH secretagogues positively correlate with the above-mentioned domains of cognitive function including attention and short-term memory (Quik et al., 2012a). Focus was then extended to comparing the effect of GHRH treatment on healthy older adults versus those suffering from mild cognitive impairment (MCI). In accordance with previous work, GHRH treatment enhanced cognitive outcomes in healthy aged individuals following a 20-week trial (Baker et al., 2012). MCI patients also displayed comparable improvements in cognitive function, however, it should be noted that these individuals did not reach non-MCI levels. Similar findings were also found in aged MCI individuals following a 20-week, self-administration treatment of the GHRH analogue tesamorelin, with improvements in cognitive function reported (Friedman et al., 2013).

The success of GH therapy on cognitive function for GHD adults and the elderly encouraged research to be conducted on the potential of GH to help recovery from forms of neurotrauma including stroke, TBI and whole brain irradiation-induced injury and will be briefly summarised below.

### Stroke

Several experimental stroke models have been designed to test the effectiveness of neuroprotection or cognitive recovery paradigms before translation to human subjects. One of the most common of these is the induction of a hypoxic-ischaemic stroke *via* carotid artery ligation. Stroke severity can be increased by lengthening the time animals are exposed to hypoxic conditions following carotid artery ligation, enabling careful control of stroke severity. Initial studies by Scheepens et al. (1999) used this approach to examine the distribution of GH receptor immunoreactive cells following injury. These investigators then determined the effect of direct ICV injection of recombinant rat GH (rrGH) in adult rats following moderate to severe stroke (Scheepens et al., 2001). Interestingly, there was a time- and severity-dependent increase in GH receptor immunoreactive cells within regions of damage. These regions included the hippocampus, the cortex and the walls of the lateral ventricles where GH receptor immunoreactivity was predominantly localised to neurons. More severe stroke samples showed more intense staining earlier. Direct ICV injection of rrGH, delivered 2-h post stroke, significantly reduced neuronal cell loss in several regions of the brain, including the cortex and hippocampus. This finding shows that GH treatment is neuroprotective in regions known to express GHR (Scheepens et al., 2001). Delaying ICV delivery of GH in rats to 4-days post stroke resulted in only moderate improvements in some measures of motor function and in spatial learning as measured by the MWM, a cognitive paradigm known to rely on the hippocampus (Pathipati et al., 2009). This diminished response of outcomes following a delay in GH supplementation may indicate that GH treatment is required immediately following injury in order to minimise tissue loss and serve as a neuroprotective agent, likely through activation of endogenous NSCs. More recently, a photothrombotic occlusion stroke model in adult mice revealed that a 28-day GH treatment, achieved *via* sub cutaneous osmotic pump delivery, resulted in reduced neural tissue loss and significant improvement in the hippocampal-dependent paired associate learning task and motor function (Ong et al., 2018). Moreover, chronic hypoxia induced hippocampal GH mRNA and protein expression while GH administration induced EPO, VEGF, and IGF-1 (Li R. C. et al., 2011). Moving to human patients, a recent trial examined the effect of treating stroke patients suffering from cognitive deficits with rhGH for a period of 6-months (Feng et al., 2020). The rhGH group displayed significant improvements (measured by the Montreal Cognitive Assessment score) relative to placebo controls, providing the tantalising prospect of utilising GH treatment in post stroke cognitive recovery. Similarly, exercise has been demonstrated to improve cognition post stroke. We also recently demonstrated improved hippocampal outcomes after 21-days of voluntary exercise in young adult mice following an endothelin-induced stroke (Codd et al., 2020), a period of exercise shown to increase circulating GH levels in young adult mice (Blackmore et al., 2021).

### Traumatic brain injury

With an ageing population, TBI is becoming more prevalent with falls being the leading cause of TBI in aged individuals (Thompson et al., 2006). For the purpose of this discussion, TBI, refers to the acquisition of a sudden trauma that causes damage to the brain. It is interesting to note that following TBI, many patients suffer from some form of pituitary dysfunction (Bondanelli et al., 2007; Devesa et al., 2013). Indeed, similar to aged individuals in whom successful induction of GH secretion correlates with increased cognitive function (Quik et al., 2012a), induced GH secretion levels can also predict positive outcomes and recovery following TBI (Bondanelli et al., 2007). To examine the prospective role of GH on cognitive recovery following TBI, a pilot study examined GHD TBI patients following a 12-month GH replacement therapy period and found significant improvement in several cognitive domains relative to placebo controls (High et al., 2010). These findings were replicated soon after (Reimunde et al., 2011) and a third study noted that the greatest improvements in GHD TBI patients occurred in those that had the most severe deficits prior to initiation of the trial (Moreau et al., 2013). These pre-clinical findings resulted in a recommendation by the US DOD for Phase II/III trials of GH after TBI (Diaz-Arrastia et al., 2014). Animal models examining the effect of TBI also revealed that TBI causes an induction of GHD and there was improved cognitive function following GH replacement therapy, whilst also offering some mechanistic insight as to how this occurs. Inducing a cortical concussion impact (CCI) *via* an impactor resulted in roughly 50% of rats displaying reduced serum GH levels (Zhang et al., 2014). A 2-week treatment period with rhGH improved spatial learning, as measured by the MWM, and increased BDNF and TrkB mRNA levels in the hippocampus (Zhang et al., 2014). BDNF has well-established roles in learning and memory functions and the recent finding that GH directly induces BDNF expression (Martinez-Moreno et al., 2018) further illustrates the pivotal role that GH plays in neural function. Recent studies have also begun to examine novel delivery approaches of GH following injury, including delivery to the spinal cord where effective delivery is considered difficult. Indeed, there is increasing evidence that nanowired delivery of GH may offer prolonged neuroprotection and reduced spinal injury compared to traditional cannula-based delivery (Muresanu et al., 2015). Moving forward, approaches such as nanowired delivery may dramatically improve outcomes following injury and is worthy of continued investigation.

### Irradiation

Cranial radiation therapy is often used in the treatment of patients suffering from a variety of malignant diseases of the brain (Barlind et al., 2010). Unfortunately, whole brain irradiation frequently causes GHD in patients (Ogilvy-Stuart et al., 1994) and a subsequent manifestation of neurocognitive dysfunction (Quik et al., 2012b). The severity of GHD appears to be related to both the dose of radiation received (Merchant et al., 2002) and the age of the patient at time of treatment, with younger patients displaying greater cognitive deficits (Chin and Maruyama, 1984). Pharmacological induction of GH secretion using GHRH in irradiation-induced GHD patients indicates that the main contributing factor of irradiation-induced GHD is hypothalamic dysregulation rather than ablation of the pituitary gland (Ogilvy-Stuart et al., 1994; Darzy and Shalet, 2006).

Animal models have also clearly demonstrated that irradiation has severe effects on the brain with greater effects apparent in developing brains. These effects include increased rates of apoptosis, greater loss of progenitor cells and a prolonged decrease in proliferation rates within the neurogenic regions (Fukuda et al., 2005). As in humans, the severity of irradiation-induced damage in animal models is dose-dependent (Mizumatsu et al., 2003). Mechanistically, it is now well known that endogenous NSCs in both the SVZ and hippocampus are exquisitely sensitive to irradiation, especially those that are mitotically active (McGinn et al., 2008). There is an increase in apoptosis (Peissner et al., 1999) and a reduction in neurogenesis rates in both the SVZ (Hellstrom et al., 2009) and dentate gyrus (Mizumatsu et al., 2003), with the dentate gyrus appearing to be more severely affected than the SVZ long-term (McGinn et al., 2008; Hellstrom et al., 2009). The consequences of irradiation-induced neural damage culminate in cognitive impairments, including spatial learning deficits (Madsen et al., 2003; Raber et al., 2004; Rola et al., 2004). These likely contribute to the observed severity of cognitive dysfunction within younger patients (Chin and Maruyama, 1984).

We previously demonstrated that direct ICV infusion of GH resulted in long-term activation of NSCs as measured by neurosphere number (Blackmore et al., 2012b). However, irradiation of animals *after* GH treatment resulted in incomplete regeneration of the SVZ, similar to that observed following a double dose of irradiation. This suggests that the GH-activated NSCs are more sensitive and vulnerable to irradiation. GH treatment *following* irradiation however, resulted in an augmented recovery of SVZ precursor cell numbers beyond non-irradiated levels (Blackmore et al., 2012b), indicating that GH can be effectively utilised in neural recovery following irradiation treatment. Similarly, Barlind et al. (2010) found that irradiated mice treated with the GH secretagogue hexarelin demonstrated an augmented hippocampal neurogenesis response.

Concerns regarding GH treatment in relation to the potential re-occurrence of malignant tumours has likely limited research in the use of GH treatment following irradiation therapy. Alternatives have therefore been investigated to aid in neural recovery following irradiation-induced damage, including physical exercise. Multiple groups have demonstrated that voluntary exercise has augmented neural recovery following irradiation-induced damage including increased neural precursor cell proliferation (Blackmore et al., 2012b), elevated neurogenesis levels (Clark et al., 2008; Naylor et al., 2008; Wong-Goodrich et al., 2010) and improved spatial learning (Wong-Goodrich et al., 2010). Considering the established role that physical exercise has in elevating GH levels, it may be an attractive alternative to GH replacement following irradiation induced damage in human subjects.

## Conclusion

**FIGURE 1:**
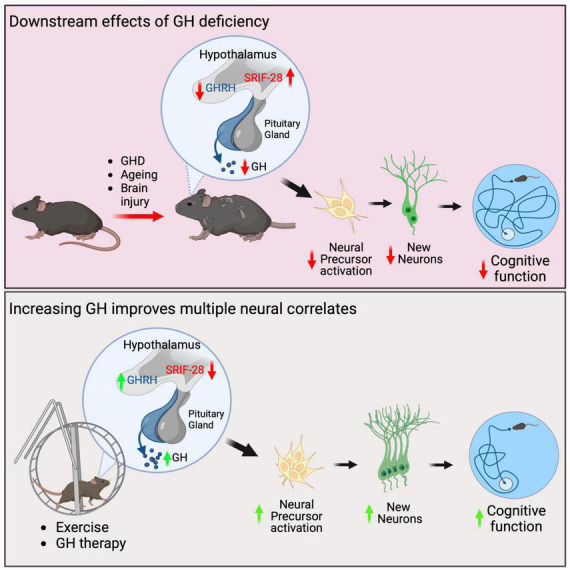
Schematics summarising the neural effects due to GHD and following GH replacement therapy in mice. GHD due to ageing or brain injury can result in hypothalamic dysfunction where GHRH is decreased and somatostatin, including somatostatin-28 (SRIF-28) is increased. This results in a decrease in GH secretion from the pituitary gland. Downstream of this reduction is a decrease in endogenous neural precursor cell activation, a decrease in neurogenesis and deficits in cognitive function, including spatial learning. Increasing GH levels either through physical exercise or GH therapy increases neural precursor cell proliferation, increases neurogenesis and improves cognitive function.

The known roles of GH have rapidly evolved. It is now clear that GH plays a critical function in the brain. Through the use of animal models, it has been possible to interrogate the complex mechanisms involved in GH-mediated changes within the brain. This includes brain development where GH aids in the proliferation of NSCs. In the adult brain, exogenous GH can activate otherwise quiescent endogenous NSCs, giving rise to increased neuron number within key regions of the brain, particularly the hippocampus. Even in ageing and following injury, GH supplementation has now been clearly demonstrated to improve cognitive outcomes. These steps and the contribution that GH plays in neural function is summarised in Figure 1. Identifying changes in the regulation of GH release allows for refinement in treatment strategies for those suffering from GHD, be it genetic or due to ageing or injury. Ultimately, the knowledge gained from these studies will allow for the tailoring of strategies to minimise side-effects and maximise treatment outcomes. With a worldwide ageing population and a concomitant increase in age-related cognitive deficits, continued research in this field is essential in order to minimise the impact for people now and in the immediate future.

## Author contributions

Both authors listed have made a substantial, direct, and intellectual contribution to the work, and approved it for publication.
